# Stratification of COVID-19 Patients with Moderate-to-Severe Hypoxemic Respiratory Failure for Response to High-Flow Nasal Cannula: A Retrospective Observational Study

**DOI:** 10.3390/medicina60010071

**Published:** 2023-12-29

**Authors:** Gianluca Bagnato, Egidio Imbalzano, Carmelo Ioppolo, Daniela La Rosa, Marianna Chiappalone, Alberta De Gaetano, Valeria Viapiana, Natasha Irrera, Veronica Nassisi, Maria Concetta Tringali, Emanuele Balwinder Singh, Nicola Falcomatà, Vincenzo Russo, William Neal Roberts, Pierpaolo Di Micco, Antonio Giovanni Versace

**Affiliations:** 1 Department of Clinical and Experimental Medicine, University of Messina, 98100 Messina, Italy; gbagnato@unime.it (G.B.); egidio.imbalzano@unime.it (E.I.); m.iop91@gmail.com (C.I.); larosa-daniela@libero.it (D.L.R.); marianna.chiappalone@gmail.com (M.C.); albi.degaetano@gmail.com (A.D.G.); nirrera@unime.it (N.I.); veronica.nassisi@gmail.com (V.N.); mariaconcetta.tringa@gmail.com (M.C.T.); emanuelesingh.polime@gmail.com (E.B.S.); nicola.falcomata@unime.it (N.F.); agversace@unime.it (A.G.V.); 2 Department of Medical Translational Sciences, Division of Cardiology, Monaldi Hospital, University of Campania “Luigi Vanvitelli”, 80138 Naples, Italy; v.p.russo@libero.it; 3 Department of Medicine, University of Kentucky, Lexington, KY 40506, USA; neal.roberts@uky.edu; 4 Emergency Department, Rizzoli Hospital, Health Authority NA2, 80122 Napoli, Italy

**Keywords:** COVID-19, HFNC, CPAP, mortality, NIV, NIRS

## Abstract

*Background and Objectives:* In patients with COVID-19, high-flow nasal cannula (HFNC) and continuous positive airway pressure (CPAP) are widely applied as initial treatments for moderate-to-severe acute hypoxemic respiratory failure. The aim of the study was to assess which respiratory supports improve 28-day mortality and to identify a predictive index of treatment response. *Materials and Methods:* This is a single-center retrospective observational study including 159 consecutive adult patients with COVID-19 and moderate-to-severe hypoxemic acute respiratory failure. *Results:* A total of 159 patients (82 in the CPAP group and 77 in the HFNC group) were included in the study. Mortality within 28 days was significantly lower with HFNC compared to CPAP (16.8% vs. 50%), while ICU admission and tracheal intubation within 28 days were significantly higher with CPAP compared to HFNC treatment (32% vs. 13%). We identified an index for survival in HFNC by including three variables easily available at admission (LDH, age, and respiratory rate) and the PaO_2_/FiO_2_ ratio at 48 h. The index showed high discrimination for survival with an AUC of 0.88, a negative predictive value of 86%, and a positive predictive value of 95%. *Conclusions:* Treatment with HFNC appears to be associated with greater survival and fewer ICU admission than CPAP. LDH, respiratory rate, age, and PaO_2_/FiO_2_ at 48 h were independently associated with survival and an index based on these variables allows for the prediction of treatment success and the assessment of patient allocation to the appropriate intensity of care after 48 h. Further research is warranted to determine effects on other outcomes and to assess the performance of the index in larger cohorts.

## 1. Introduction

Coronavirus disease 2019 (COVID-19) is an infectious disease induced by severe acute respiratory syndrome coronavirus 2 (SARS-CoV-2) [1] with heterogeneous clinical manifestations, ranging from asymptomatic infection to severe forms of respiratory failure defined as acute respiratory distress syndrome (ARDS) in about 15% of the cases [2,3,4,5,6,7,8]. 

This latter condition requires intensive respiratory support and is associated with a higher risk of mortality [9,10,11,12,13,14,15,16].

Currently, in the management of ARDS, in the absence of concomitant chronic obstructive pulmonary disease (COPD) or pulmonary edema, the benefits of non-invasive ventilatory strategies, either CPAP or bilevel positive airway pressure (BiPAP), are controversial [17,18,19,20]. In fact, some guidelines propose non-invasive ventilatory strategies as an alternative in the absence of high-flow nasal cannula (HFNC) and others only in the presence of hypercapnia [21,22]. In the systematic review and meta-analysis by Cammarota et al., patients admitted for COVID-19 and requiring non-invasive ventilatory support outside the ICU were characterized by an overall intra-hospital mortality of 36% [23], substantially higher than the intra-hospital mortality observed in the group treated with non-invasive ventilatory support from a recent randomized-controlled trial conducted in COVID-19 patients admitted to ICU, whereas 24% in the helmet CPAP group and 25% in the HFNC group died [24]. 

In the RECOVERY-RS multicenter, in a randomized clinical trial among patients with acute hypoxemic respiratory failure due to COVID-19, an early approach with CPAP significantly decreased the risk of tracheal intubation compared to conventional oxygen therapy, whereas an initial strategy of HFNC did not significantly differ from standard oxygen therapy [25]. This is in contrast with a retrospective study and another randomized clinical trial that reported reduced rates of intubation and need for mechanical ventilation in patients treated with HFNC compared to other modalities [26,27]. Nevertheless, the criteria for starting non-invasive ventilatory strategies in patients with moderate-to-severe respiratory failure remains debatable and warrants larger head-to-head studies [28,29]. One of the key challenges, according to the conflicting evidence reported in the management of severe hypoxemic acute respiratory failure in COVID-19 patients, is therefore an appropriate patient stratification and the identification of predictors of response to non-invasive strategies.

Due to the existing conflicting evidence and the relevant unmet clinical needs, the aim of this study was to accurately predict HFNC response in order to offer the possibility to allocate COVID-19 patients with ARDS to HFNC treatment and to verify that clinical choice at 48 h, thereby increasing high-intensity care units’ bed availability for more advanced respiratory support. Thus, in this ascertainment study, we aimed first to retrospectively identify an early predictive index to stratify COVID-19 patients with moderate-to-severe acute hypoxemic respiratory failure for continuance of HFNC rather than CPAP. A subsequent series including patients excluded from the ascertainment set here will be required to validate the index for its envisioned use at the moment of admission for all patients.

## 2. Materials and Methods

### 2.1. Study Population

In this single-center retrospective observational study, we included 375 consecutive adult patients (≥18 years of age) with a laboratory-confirmed COVID-19 infection associated with acute hypoxemic respiratory failure and/or ARDS defined by Berlin criteria [30], treated with HFNC or CPAP.

Recruited patients were admitted to the COVID-19 medical emergency department of the University of Messina between March 2021 and October 2022. Consent was obtained from the patients to use and publish their data. The study was approved by the ethics committee of the hospital (Prot. N. 62-22). All included patients had acute hypoxemic respiratory failure with peripheral oxygen saturation (SpO^2^) < 92% despite conventional low-flow oxygen therapy and PaO_2_/FiO_2_ < 200. 

Exclusion criteria were the transition in our division for direct admission to the intensive care unit for endotracheal intubation, unconsciousness or drowsiness, the use of non-invasive ventilation, the absence of data regarding respiratory management, and patients with a do not intubate order. Patients who received CPAP or HFNC for less than 12 h and therefore were treated with conventional oxygen therapy (COT) at the time of screening were also excluded.

### 2.2. Data Collection

Data were collected from the electronic health records including, admission vital signs, clinical presentation, laboratory measurements, and outcome of interest (mortality). Recorded data also included demographics (age, gender, body mass index (BMI), number of comorbidities, Charlson Comorbidity Index (CCI)) [31], disease chronology (time from onset of symptoms and from hospital admission to initiation of respiratory support, length of stay), vital signs (temperature, mean arterial pressure, heart rate), laboratory parameters (blood test, coagulation, biochemical), ratio of oxygen saturation to inspired oxygen fraction, divided by respiratory rate index (ROX), SpO_2_/FiO_2_, and PaO_2_/FiO_2_. These data were collected for all patients. After placement of HFNC or CPAP, arterial blood gas analysis parameters, SpO_2_/FiO_2_, PaO_2_/FiO_2_, and ROX follow-up scores were again collected at 6, 24, and 48 h from admission. 

### 2.3. Respiratory Support

HFNC is endorsed as the standard treatment for patients with COVID-19 failing on conventional low-flow oxygen therapy in both local guidelines and current international guidelines. HFNC (Optiflow^TM^ nasal high-flow interface) was provided by the AIRVO 2 humidification system (Fisher and Paykel, Rome, Italy). The flow was set from 30 to 60 L/min according to patient respiratory rate. The fraction of inspired oxygen (FiO_2_) was adjusted to reach a peripheral blood oxygen saturation (SpO_2_) above 94% [32].

CPAP was used according to evidence, to clinical practice, and to physician judgment. We used a (DIMAR, Cuneo, Italy) full-face mask or (DIMAR, Italy or Starmed-Intersurgical, Rugby, UK) helmet driven by high-flow blender, according to local availability. The full-face mask size was chosen according to the measuring tape provided by DIMAR, and helmet size was chosen according to neck circumference. CPAP was started at a FiO_2_ of 40% which could be increased according to physician judgement. PEEP of 5 cmH_2_O could be increased to a maximum of 25 cmH_2_O. Oxygen settings were titrated to a recommended target SpO_2_ around 94–96%. In case of intolerance to CPAP, we used HFNC as a rescue, if the condition did not necessitate urgent endotracheal intubation. When the clinical status and parameters (vital signs, hemodynamics, blood gas exchange, and respiratory rate) showed signs of improvement, we applied intermittent use of HFNC and CPAP, and then HFNC only until complete weaning [33].

### 2.4. Study Outcome

The outcomes aimed to define the superiority of advanced respiratory support, according to 28-day mortality rate, and to identify a predictive index of treatment response at admission and after 48 h of observation. 

### 2.5. Statistical Analysis

Continuous data were summarized using the number of participants (percentage), mean (SD or 95% CI), and median (IQR). Categorical data were summarized with frequency count and percentage. Odds ratios (95% CIs) were reported for categorical outcomes using logistic regression models. Mean or median differences (95% CIs) were reported for continuous outcomes. Proportional (absolute) differences (95% CIs) were reported for categorical outcomes. Continuous variables were compared with the *t* test with unequal variances or the Mann–Whitney U test, as appropriate. Categorical variables were compared using the chi-square tests or Fisher’s exact test as appropriate. We reported odds ratios (OR) with their associated 95% confidence intervals (CI). Univariable logistic regression analysis was performed for all the variables that were significant in the between-group analysis. The change in risk for a predictor variable in a logistic regression model was calculated according to the following formula: (Odd ratio − 1) × 100. To enhance the pragmatic approach, the significant variables were plotted in their categorized form, according to the reference range of the test (present/absent), or in a continuous form, in a multivariable logistic regression analysis with the backward stepwise method. During the derivation step, all variables that showed statistical significance with the outcome were chosen, and a final model was fitted based on the best accuracy. The area under the curve (AUC) for the predictive model was displayed using a receiver operating characteristic (ROC) curve. The optimal cut-off was chosen as the one with the highest accuracy maximized for sensitivity and specificity. The model’s performance is reported as sensitivity, specificity, positive and negative predictive values, and their 95% CI [34]. All results with 2-sided *p* ≤ 0.05 were considered statistically significant. Statistical analysis was performed with SPSS (version 22, IBM, Armonk, NY, USA).

## 3. Results

### 3.1. Demographic, Clinical, and Respiratory Characteristics According to Respiratory Support Allocation at Admission 

From March 2021 to October 2022, 375 critically ill patients with COVID-19-related acute hypoxemic respiratory failure were included in the present study. From those, 216 were excluded: 11 patients had incomplete data, 175 patients were stable with conventional oxygen therapy, 29 patients were initially treated with NIV, and a “do not intubate order” was present in 12 patients. A total of 159 participants (82 in the CPAP group and 77 in the HFNC group) were then included in the study (Supplementary Figure S1). Initially, we analyzed the demographic, clinical, laboratory, and respiratory profile at admission of the study population according to the mortality rate (Supplementary Tables S1 and S2). Accordingly, the study population was divided into deceased (*n* = 54; 33.9%) and survivors (*n* = 105; 66.1%). Next, we compared the significantly different variables between the groups in a univariate logistic regression and the results showed that age, Charlson Comorbidity Index, SO_2_, SpO_2_, CPAP treatment, LDH, neutrophil-to-lymphocyte ratio, and respiratory rate at admission were associated with mortality (Supplementary Table S3). These variables were analyzed in a multivariate analysis and the model identified age and CPAP treatment as the factors independently associated with mortality in our study population (Supplementary Table S4).

Because CPAP treatment was associated with a negative outcome, we divided our study population according to the respiratory support in HFNC (*n* = 77) and CPAP (*n* = 82). Demographic characteristics, vital parameters, clinical features, and comorbidities at admission for the group of HFNC patients (*n* = 77) and CPAP patients (*n* = 82) are shown in Table 1. Participant characteristics were similar at baseline, with no differences in gender prevalence, age, or body mass index (BMI) across the groups, apart from a longer hospital stay and lower mortality and intubation rate in the HFNC group.

Next, we compared the laboratory results between the groups and we observed that HFNC was associated with lower levels of IL-6, the neutrophil-to-lymphocyte ratio, and LDH (Table 2). 

After the evaluation of the demographic, clinical, and laboratory results, we compared respiratory parameters at admission between the groups. The respiratory parameters reported in Table 3 refer to conventional oxygen therapy at admission before treatment with either HFNC or CPAP. Among the data obtained from arterial blood gas analysis, both pO_2_ and SO_2_ were significantly higher in the HFNC group compared to the CPAP group.

Among the respiratory indexes, the ROX score and SpO_2_ were higher in the HFNC group while the respiratory rate was lower in the group of patients treated with HFNC. No significant differences were observed in PaO_2_/FiO_2_ and SpO_2_/FiO_2_ values between the groups. 

After the assessment at admission, arterial blood gas analysis and respiratory indexes were analyzed at 6, 24, and 48 h. During the observation period, in the HFNC group, the median flow was 60 L (IQR 50–60) and the median FiO_2_ was 60% (IQR 50–60), while in the CPAP group, median PEEP was 10 cmH_2_O (IQR 10–10) and the median FiO_2_ was 60% (IQR 55–60).

### 3.2. Mortality and Admission to ICU Rates in HFNC and CPAP Groups

**Figure 1:**
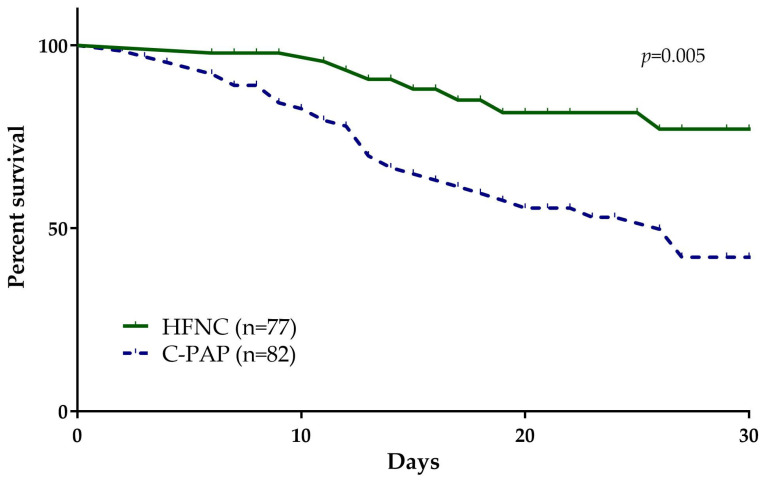
Kaplan–Meier curve for study participants treated with HFNC (green line, *n* = 77) and CPAP (blue line, *n* = 82). Patients treated with HFNC have a significantly higher percent survival compared to patients treated with CPAP (*p* = 0.005).

Next, we analyzed the 28-day mortality rate between the groups and we observed that it occurred in a significantly higher proportion of patients treated with CPAP compared to HFNC (50%, *n* = 41/82 vs. 16.8%, *n* = 13/77; *p* = 0.005; Figure 1). Regarding the ICU admission, there was a significant difference in the individual incidence of admission to ICU and tracheal intubation within 28 days (Table 2).

### 3.3. Predictors of Response to HFNC at Admission

Due to the significant difference according to the 28-day mortality rate and in order to identify the predictors of response to HFNC, patients were further stratified into two subgroups according to the outcome of interest: HFNC survivors, including only patients treated with HFNC that survived, and CPAP failure, including only patients treated with CPAP that died.

The comparison of demographic characteristics, vital parameters, clinical features, and comorbidities at admission between the HFNC survivors (*n* = 64) and CPAP failure (*n* = 41) groups is shown in Supplementary Table S5.

Patients in the HFNC survivors group were significantly younger than the other group with longer hospital stay. Additionally, there was a higher incidence of ICU admission and tracheal intubation within 28 days in the CPAP failure group (60.9% vs. 4.62%; *p* = 0.001). 

Next, we compared the laboratory results between the HFNC survivors and CPAP failure groups (Supplementary Table S6). Our results showed that IL-6, LDH, neutrophil-to-lymphocyte ratio, and troponin serum levels were significantly higher in the CPAP failure group, while albumin serum levels were significantly lower compared to the HFNC survivors group. 

First, we focused the progression of the results on the relevant variables at admission.

After identifying the factors that were statistically different between HFNC survivors and CPAP failure, univariable logistic regression analysis was performed, showing that age, IL-6, LDH, neutrophil-to-lymphocyte ratio, and respiratory rate at admission were independent predictors of HFNC survival (Table 4).

Next, we analyzed the results of the univariate analysis in a multivariate analysis. The following variables were retained by the model: respiratory rate at admission, age, and LDH (Table 5).

### 3.4. HFNC Survival Index at Admission

After the identification of the variables independently associated with HFNC survival, a model was developed using the significant admission parameters (LDH, respiration rate, and age) of the multivariable logistic regression in order to identify an index that may be applied at the time of hospital admission to stratify patients for HFNC treatment response.

**Figure 2:**
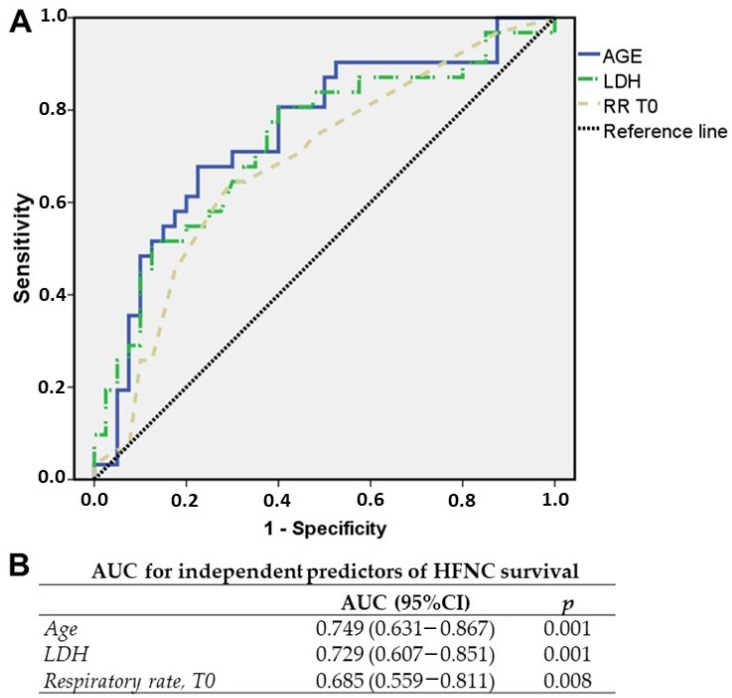
(**A**,**B**): Receiver operating characteristic curves for age, LDH, and respiratory rate at admission (T0) for HFNC survival at admission. The area under the curve (AUC) for each variable is reported in (**B**). RR T0 = respiratory rate at admission.

These variables were plotted in an ROC curve and the AUC was calculated for each variable (Figure 2A,B). After sensitivity and specificity maximization, we identified the cut-off value for the best predictivity performance. Once the value for each parameter was determined (LDH ≤ 438; age ≤ 77.28; respiratory rate at admission ≤ 23), these were classified in a binary format (LDH ≤ 438 = 1; age ≤ 77.28 = 1; respiratory rate at admission ≤ 23 = 1).

**Figure 3:**
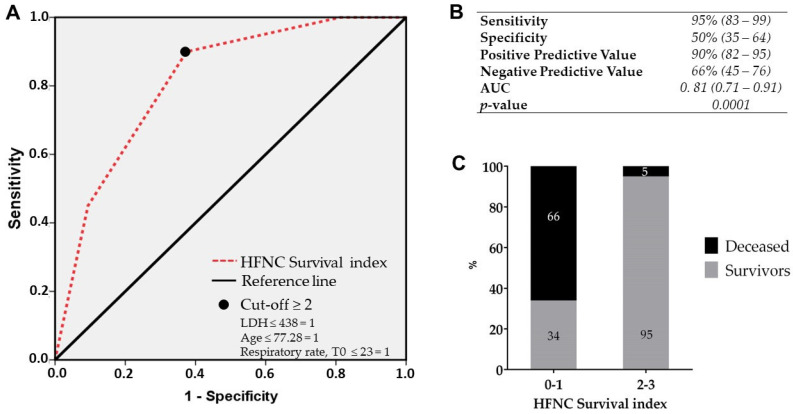
(**A**) shows the receiver operating characteristic (ROC) curves for the performance of the HFNC survival index at admission. Sensitivity, specificity, and positive and negative predictive values according to the reference cut-off are reported in (**B**). The performance of this score applied in the entire HFNC population is reported in (**C**), categorized according to the cut-off value of the HFNC survival index. Respiratory rate, T0 = respiratory rate at admission.

As reported in Figure 3A,B, a score ≥2 showed the best performance with an AUC of 0.81 (95% CI: 0.71–0.91) and allowed us to identify the HFNC survival index with a sensitivity of 95% (95% CI: 83–99), specificity of 50% (95% CI: 35–64), a negative predictive value of 66% (95% CI: 45–76), and a positive predictive value of 90% (95% CI: 82–95) *p* = 0.0001.

Next, the performance of this index was tested only in the entire group of patients treated with HFNC from the original cohort. The application of this index in the entire group of patients treated with HFNC showed an excellent performance: 95% of the patients treated with HFNC having a score of ≥2 survive, and oppositely, 66% of patients treated with HFNC with a score ≤1 will not respond to HFNC (Figure 3C). Thus, among 64 HFNC survivors, the score proved to be able to predict a favorable outcome in 61 patients, while among 13 patients that deceased under HFNC treatment, the score was able to identify 9 patients.

### 3.5. HFNC Survival Timing Index at 48 h

Next, we decided to improve the performance of the HFNC survival index based on admission parameters by including a time-dependent variable. The following respiratory parameters measured over time were compared between the groups for each time point considered (6 h, 24 h, and 48 h): respiratory rate, ROX, PaO_2_/FiO_2_, and SpO_2_/FiO_2_ (Table S7). The variables showing a significant difference between the groups were then analyzed separately for each time point by univariate analysis first (Table 4) and then by multivariate analysis (Table 5). 

According to the multivariate analysis, one variable resulted independently associated with HFNC response in the HFNC survivors group compared to CPAP failure for each timepoint: ROX score at 6 h, ROX score at 24 h, and PaO_2_/FiO_2_ at 48 h (Table 5).

Next, these three factors were analyzed in a multivariate logistic regression and PaO_2_/FiO_2_ at 48 h was retained by the model as the single variable significantly associated with HFNC survival (OR 1.031, 95% CI 1.011–1.052, *p* = 0.003).

These three variables were then plotted in an ROC curve for confirmatory purposes and the AUC for the independent respiratory predictors over time for HFNC survival was calculated using the univariate logistic regression results.

Indeed, PaO_2_/FiO_2_ at 48 h showed the best performance according to the ROC curve and AUC (Supplementary Figure S2). Thus, PaO_2_/FiO_2_ at 48 h was included in the index based on the results of the AUC and the multivariate logistic regression.

Following sensitivity and specificity maximization, the cut-off value for PaO_2_/FiO_2_ at 48 h was identified (PaO_2_/FiO_2_ ≥ 121) and categorized in binary format. This variable was included in the HFNC Survival index to provide the best four parameters for the final time-improved index. After applying the cut-off for each variable (LDH ≤ 438, age ≤ 77.28, respiratory rate at admission ≤ 23, and PaO_2_/FiO_2_ at 48 h ≥ 121), these were categorized in a binary format, with a cut-off value of ≥3. The resulting HFNC Survival Timing index showed a very good performance with an AUC of 0.88 (95% CI: 0.80–0.96), sensitivity of 95% (95% CI: 83–99), specificity of 87% (95% CI: 67–95), positive predictive value of 97% (95% CI: 85–99), and negative predictive value of 77% (95% CI: 61–89).

**Figure 4:**
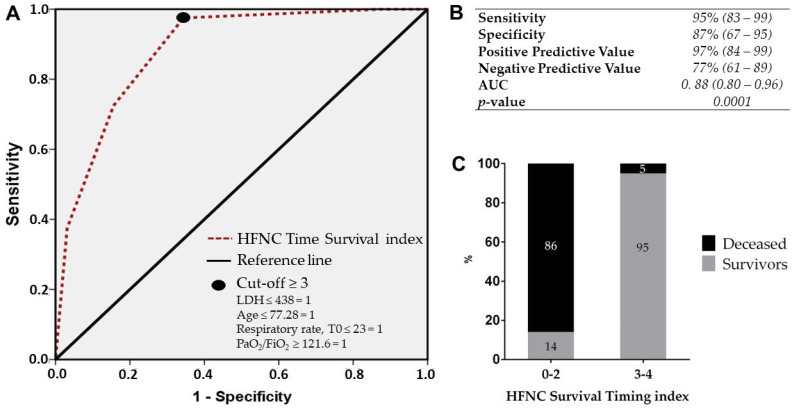
(**A**) shows the receiver operating characteristic (ROC) curves for the performance of the HFNC Survival Timing index. Sensitivity, specificity, and positive and negative predictive values according to the reference cut-off are reported in (**B**). The performance of this index applied in the entire HFNC population is reported in (**C**), categorized according to the cut-off value. Respiratory rate, T0 = respiratory rate at admission.

Again, the index was applied only in the entire group of patients treated with HFNC to verify its performance both in survivors and deceased patients treated with HFNC. The HFNC Survival Timing index showed an improved ability to identify HFNC failure (86% vs. 66%, Figure 4C) compared to the HFNC survival index. Indeed, among 64 HFNC survivors, the score proved to be able to predict a favorable outcome in 61 patients, while among 13 patients that died under HFNC treatment, the score was able to identify a higher number of patients (11 vs. 9).

## 4. Discussion

In our study, we preliminarily compared mortality rate according to respiratory support allocation between HFNC and CPAP. Because the mortality rate was lower in the group of COVID-19 patients treated with HFNC, we identified a score, defined as the HFNC survival index, for HFNC response, based on LDH values (cut-off value < 438), age (cut-off value > 77.28), and respiratory rate (cut-off value < 23) at admission, to identify candidates for an early strategy of HFNC rather than CPAP. The negative predictive value of the HFNC survival index was further improved by including PaO_2_/FiO_2_ at 48 h from admission (cut-off value > 121.6), to provide a confirmatory time-dependent variable that composes the final HFNC time survival index.

Indeed, some studies suggest that HFNC, a relatively new technique that is easy to use and employed also in non-ICU settings, is a better approach to start with for the treatment of mild-to-moderate respiratory failure in COVID-19 patients [21,29,35,36,37], even in those with a do not intubate order [38]. However, this option does not appear to be applicable to the entire population of COVID-19 patients with moderate-to-severe hypoxemic respiratory failure. Thus, our results aim to provide a tool to stratify for HFNC response at admission and to start the appropriate respiratory support with HFNC according to the probability of response without switching also in COVID-19 patients with more severe respiratory failure.

To date, the use of HFNC and CPAP in COVID-19 patients has not been compared directly in any trial. The HELMET-COVID Randomized Clinical Trial was the only one designed to compare helmet CPAP versus HFNC in COVID-19 patients with respiratory failure, but the results were never published as the study was not concluded due to difficulties in recruiting patients at study start because full-face masks replaced the helmet interface as the local non-invasive respiratory support (NIRS) standard [39]. 

The RECOVERY-RS randomized clinical trial evaluated the use of CPAP and HFNC in comparison to the identical control group receiving standard oxygen therapy in a study that was essentially conducted as two distinct trials. In comparison to the group receiving conventional oxygen therapy, the proportion of patients in the CPAP arm who met the primary endpoint was considerably lower. However, this difference was completely attributable to a decrease in the number of patients who required intubation, not to death rate. Also, in this case, a direct comparison between HFNC and CPAP was not implemented in the study design. Furthermore, the RECOVERY-RS trial was terminated for reasons unrelated to futility, efficacy, or harm long before the intended sample size was attained; hence, drawing conclusions about benefit in this situation may be difficult [25]. 

Additionally, HFNC was compared to standard oxygen treatment in 220 patients with severe COVID-19 recruited in three Colombian hospitals in the High-Flow Nasal Cannula in the Severe COVID-19 With Acute Hypoxemic Respiratory Failure (HiFLo-COVID) randomized clinical trial. HFNC reduced the need for tracheal intubation (hazard ratio, 0.62; 95% confidence interval, 0.39–0.96) and the time to clinical recovery, but it had apparently no effect on mortality [27]. 

In the HENIVOT Randomized Clinical Trial, 109 patients with moderate-to-severe COVID-19 respiratory failure were randomly assigned to receive HFNC or NIRS via a helmet device. When comparing the arms for the main outcome, days without breathing support, the study revealed no differences between the arms. However, while 51% of patients in the HFNC oxygen arm needed endotracheal intubation, 30% of patients in the NIRS arm required it [24].

Therefore, the relevant therapeutic question is whether to employ CPAP or HFNC in certain circumstances and whether some patients with specific characteristics may benefit from one support over another to allocate the appropriate respiratory support to the right patient [40,41]. Indeed, recent trials on CPAP in COVID-19 patients with ARDS, despite lacking a head-to-head design, are improving the selection and stratification criteria and providing further promising data on the safety profile [42,43,44,45,46].

According to several reports, COVID-19-related ARDS is a result of differences between peripheral saturation, lung compliance, blood gas, and the incidence of systemic consequences [47]. Due to the fact that patients with COVID-19 pneumonia present with reduced PaO_2_ values while maintaining adequate SpO_2_, SpO_2_ alone may be misleading in determining the progression of the disease. This condition is referred to as “happy” or “silent” hypoxia, which is characterized by a markedly elevated respiratory rate, and, unlike hypercapnia, does not appear to induce dyspnea, and in turn induces the clinician to underestimate the severity of the disease, delaying the clinical choice on admission allocation, and/or postpone urgent treatment [11,12,13,14,48].

Indeed, our simple model takes into account respiratory rate, together with LDH, age, and PaO_2_/FiO_2_, as one of the four relevant variables in composing the predictive index of HFNC survival. Among these, LDH levels have been observed to be elevated during acute and severe lung injury, and elevated LDH levels have been found in different interstitial lung infections [49]. LDH may be an expression of lung injury progression or a marker of disease activity in COVID-19 patients, reflecting respiratory discomfort caused by the aberrant inflammatory condition. Additionally, LDH has been related to respiratory function (PaO_2_/FiO_2_) and is considered a predictor of respiratory function worsening in COVID-19 patients [50,51]. The introduction of PaO_2_/FiO_2_ at 48 h further improves the negative predictive value of the HFNC survival index based on admission variables. Indeed, this is confirmed by other reports suggesting that the risk of HFNC is reduced after 48 h exposure, providing the timing to separate HFNC responders to non-responders [52]. One of the main criteria to escalate to invasive strategies in non-COVID-19 critically ill patients has been the PaO_2_/FiO_2_ value [53,54]. On the other hand, as reported by Mellado-Artigas et al. in a multicenter observational study, PaO_2_/FiO_2_ does not appear to be equally useful in the assessment of intubation risk in COVID-19 patients [55]. These findings further support the acquisition of new data for the prediction of response to different respiratory devices in COVID-19 patients compared to other ARDS patients, due to specific alterations inducing anatomic and functional shunts [56,57]. Nonetheless, our findings provide a target PaO_2_/FiO_2_ that can confirm the stratification adopted at admission for HFNC response and survival. 

However, we acknowledge some limitations of our study. First, the retrospective design of the study does not allow us to extend the results to the general population because our data were collected from a single center without any validation in other cohorts. In addition, despite patients enrolled in the study having been infected by different SARS-CoV-2 variants, no subgroup analysis was performed due to absence of available testing for the entire cohort Also, no specific subgroup analysis according to different CPAP interfaces (helmet vs. full-face) has been performed due to unbalanced distribution. In addition, due to the design of the study that focused on respiratory parameters at admission, the patients’ classification according to nadir PaO_2_/FiO_2_ was not included. More importantly, the choice of the most appropriate device was based upon a clinical decision, response to treatment, and availability of the devices. Thus, a potential intrinsic selection bias might be strong and certainly cannot be excluded due to the retrospective study design. The strength of the index’s performance in this ascertainment set may hint that it can overcome a strong confounding by indication vis-a-vis the type of NIV treatment allocation. 

While the identification of the index was based on the arbitrary comparison between HFNC patients that survived and CPAP patients that died, the index was then applied to the entire group of patients treated with HFNC, thus providing a partial internal validation of the index itself. A subsequent series including patients excluded from the ascertainment set here will nevertheless be required to validate the index for its intended use at the moment of admission. Finally, it is important to emphasize that the principal aim of the current study was to stratify for HFNC response to offer the possibility to allocate COVID-19 patients with ARDS to HFNC treatment and to verify the clinical choice at 48 h, thus increasing high-intensity care units’ bed availability for more advanced respiratory support, because HFNC is also relatively easy to use in non-ICU wards. 

## 5. Conclusions

In conclusion, in this retrospective study of 159 adult critically ill patients with COVID-19-related acute respiratory failure, we identified the HFNC survival index based on age, LDH, and respiratory rate with its time extension that includes PaO_2_/FiO_2_ at 48 h that increases the possibility to test for responders over time. This index represents a feasible method to stratify COVID-19 patients with respiratory failure for HFNC success and offers the advantage of being easy to use, thus allowing the clinicians to allocate patients to non-ICU wards, stratify patients to the appropriate intensity of care, and test treatment response at admission and after 48 h. The HFNC time survival index might offer the possibility to guide treatment decisions and improve survival in severely ill COVID-19 patients.

## Figures and Tables

**Table 1:** Demographics and clinical parameters of the study population at admission according to respiratory support.

	HFNC (*n* = 77)	CPAP (*n* = 82)	*p*
Demographics			
Age, years	72 (63–78)	73 (64–80)	0.394
BMI	27.6 (24.4–29.4)	27.3 (24.4–29.4)	0.469
Gender, male	29 (37.6)	34 (41.4)	0.582
Hospital stay (days)	22.5 (16–29)	14 (8–19.5)	0.001
Mortality (28 days)	13 (16.8)	41 (50)	0.001
ICU admission (28 days)	10 (13)	26 (32)	0.003
COVID-19 vaccination	16 (20.7)	12 (14.6)	0.332
Comorbidities	2 (1–5)	2 (1–4)	0.408
Charlson Comorbidity Index	4 (2–6)	4 (2.25–6)	0.891
Diabetes	43 (55.8)	38 (46.3)	0.264
Coronary artery disease	25 (32.5)	22 (26.8)	0.459
COPD	16 (20.7)	12 (14.6)	0.332
Heart failure	23 (29.8)	25 (30.4)	0.891
Hypertension	43 (55.8)	54 (65.8)	0.164
Chronic kidney disease	12 (15.5)	17 (20.7)	0.381
Atrial fibrillation	11 (14.2)	10 (12.9)	0.725
Cerebrovascular disease	5 (6.4)	8 (9.7)	0.436
Respiratory failure	3 (3.8)	2 (2.4)	0.611
Vital and clinical parameters			
Heart rate	80 (70–95)	82 (76–92)	0.777
DBP mmHg	80 (65–85)	79 (70–85)	0,689
SBP mmHg	135 (125–145)	140 (130–150)	0.114
MBP mmHg	96 (85–105)	100 (93–104)	0.193
Fever (≥38 °C)	31 (40.2)	39 (47.5)	0.319
Glasgow Coma Scale	15 (15–15)	15 (15–15)	0.665
Therapy			
Corticosteroids	77 (100)	82 (100)	1
Anti-IL6	8 (10.3)	8 (9.7)	0.917
Remdesivir	2 (2.5)	2 (2.4)	0.967
Anti-SARS-CoV-2 antibody	4 (5.1)	4 (4.87)	0.954

Data are *n* (%) and median (IQR). *n* is the total number of patients with available data. BMI: body mass index; COPD: chronic obstructive pulmonary disease; COVID-19: coronavirus disease 2019; DBP: diastolic blood pressure; IL-6: interleukin 6; ICU: intensive care unit; MBP: mean blood pressure; SARS-CoV-2: severe acute respiratory syndrome coronavirus 2; SBP: systolic blood pressure.

**Table 2:** Laboratory findings profile according to the respiratory support.

	HFNC (*n* = 77)	CPAP (*n* = 82)	*p*
Laboratory findings			
Albumin, g/dL	3.13 (2.9–3.3)	3.1 (2.7–3.4)	0.582
ALT, UI/L	24.5 (15–42)	29 (19–55)	0.101
AST, UI/L	29 (20–40)	31 (23–53)	0.216
CK, U/L	100 (50–214)	83.5 (44–241)	0.950
Creatinine, mg/dL	0.9 (0.8–1.2)	0.8 (0.7–1.4)	0.147
D-Dimer, mcg/mL	3 (3–4)	4 (4–4)	0.953
Fibrinogen, mg/dL	464 (342–889)	533 (360–636)	0.595
Hb, gr/dL	14 (10–14.5)	14 (11.5–15.5)	0.620
IL-6, pg/mL	28 (9–63.9)	70 (42.7–129)	0.001
LDH, U/L	339 (276–432)	425 (334–574)	0.004
Bilirubin, total, mg/dL	0.63 (0.43–0.85)	0.57 (0.45–0.93)	0.895
NT-proBNP, pg/mL	504 (163–1712)	574 (163–2071)	0.703
CRP, mg/dL	6.3 (3.2–14.1)	10.1 (5.9–14.6)	0.119
PCT, ng/mL	0.15 (0.08–0.52)	0.12 (0.08–0.3)	0.920
Ferritin, mg/dL	642 (252–2235)	905 (480–1621)	0.519
Platelet count, cells × 10^4^	198 (106–244)	205 (151–282)	0.294
Troponin, pg/mL	32.5 (15.4–87)	28 (11.7–63.9)	0.238
Urea, mg/dL	77.5 (48.5–154.7)	61 (40.5–114)	0.772
WBC, cells	8600 (5150–15,175)	9100 (6800–13,150)	0.096
Lymphocyte count, cells	994 (657–1416)	742 (551–1081)	0.370
Neutrophil count, cells	6382 (3823–13,446)	8010 (5731–11,614)	0.126
Neutrophil/lymphocyte ratio	7.2 (3.8–11)	9.5 (6.8–15.8)	0.024

Data are *n* (%) and median (IQR). *n* is the total number of patients with available data. ALT: Alanine transaminase; AST: aspartate transaminase; CK: creatine kinase; CRP: C-reactive protein; Hb: hemoglobin; IL-6: interleukin 6; LDH: lactate dehydrogenase; NT-proBNP: N-terminal pro-brain natriuretic peptide; PCT: procalcitonin; WBCs: white blood cells.

**Table 3:** Respiratory parameters of the study population according to respiratory support.

	HFNC (*n* = 77)	CPAP (*n* = 82)	*p*
Arterial blood gas analysis			
PaO_2_	62 (56–69)	58 (54–63)	0.039
PaCO_2_	35 (33–37)	34 (32–38)	0.161
SO_2_	94 (90–95)	91 (88–93)	0.004
FiO_2_	60 (50–60)	60 (60–60)	0.564
lactates	1.45 (1–2.3)	1.6 (1.1–2.5)	0.485
Respiratory Indexes			
Respiratory Rate	20 (20–25)	25 (20–28)	0.014
SpO_2_	93 (90–95)	92 (88–94)	0.012
PaO_2_/FiO_2_ ratio	112 (97–125)	101 (92–110)	0.064
SpO_2_/FiO_2_ ratio	158 (152–179)	155 (148–160)	0.067
ROX	7.4 (6.8–8.1)	6.6 (5.4–7.8)	0.025
Berlin criteria			
PaO_2_/FiO_2_ ≤ 100	28 (36.4)	35 (42.6)	0.418
>100 PaO_2_/FiO_2_ ≤ 200	49 (63.6)	47 (57.3)	0.418

Data are *n* (%) and median (IQR). *n* is the total number of patients with available data. FiO_2_: fraction of inspired oxygen; PaCO_2_: partial pressure of carbon dioxide; PaO_2_: partial pressure of oxygen; ROX: respiratory rate oxygenation; SO_2_: Oxygen saturation.

**Table 4:** Univariable logistic regression analysis for HFNC survival at admission, at 6, 24, and 48 h.

	OR (95% CI)	Change in Risk (%)	*p*
At admission			
Age, years	0.909 (0.856–0.965)	−9.1	0.002
Albumin, g/dL			0.285
LDH, U/L	0.995 (0.991–0.999)	−0.5	0.008
Neutrophil-to-lymphocyte ratio	0.914 (0.845–0.989)	−8.6	0.025
IL-6, pg/mL	0.981 (0.968–0.994)	−1.9	0.004
Troponin, pg/mL			0.703
Respiratory rate	0.859 (0.768–0.961)	−14.1	0.008
6 h			
Respiratory rate	0.801 (0.688–0.932)	−19.9	0.004
SpO_2_/FiO_2_	1.21 (1.001–1.043)	21	0.044
ROX	1.743 (1.226–2.478)	74.3	0.002
24 h			
Respiratory rate	0.808 (0.688–0.95)	−19.2	0.010
SpO_2_/FiO_2_	1.024 (1.007–1.04)	2.4	0.005
ROX	1.436 (1.125–1.833)	43.6	0.004
PaO_2_/FiO_2_	1.013 (1.002–1.025)	1.3	0.022
48 h			
Respiratory rate			0.819
ROX	1.37 (1.06–1.77)	37	0.017
PaO_2_/FiO_2_	1.032 (1.012–1.054)	3.2	0.003

FiO_2_: fraction of inspired oxygen; IL-6: interleukin 6; LDH: lactate dehydrogenase; PaCO_2_: partial pressure of carbon dioxide; PaO_2_: partial pressure of oxygen; ROX: respiratory rate oxygenation; SO_2_: oxygen saturation.

**Table 5:** Multivariable logistic regression analysis for HFNC survival at admission, at 6, 24, and 48 h.

	OR (95% CI)	Change in Risk (%)	*p*
At admission			
Age, years	0.922 (0.866–0.977)	−7.8	0.024
CCI			0.092
LDH, U/L	0.995 (0.992–0.999)	−0.5	0.013
Respiratory rate	0.864 (0.757–0.986)	−13.6	0.030
Neutrophil-to-lymphocyte ratio			0.088
IL-6, pg/mL			0.124
6 h			
ROX	1.743 (1.226–2.478)	74.3	0.002
SpO_2_/FiO_2_			0.423
Respiratory rate			0.374
24 h			
ROX	1.429 (1.116–1.830)	42.9	0.005
SpO_2_/FiO_2_			0.734
Respiratory rate			0.840
PaO_2_/FiO_2_			0.582
48 h			
PaO_2_/FiO_2_	1.041 (1.008–1.075)	4.1	0.013
ROX			0.490

CCI: Charlson Comorbidity Index; FiO_2_: fraction of inspired oxygen; IL-6: interleukin 6; LDH: lactate dehydrogenase; PaO_2_: partial pressure of oxygen; ROX: respiratory rate oxygenation; SpO_2_: peripheral oxygen saturation.

## Data Availability

The data presented in this study are available on request from the corresponding author.

## Supplementary Materials

The following supporting information can be downloaded at https://www.mdpi.com/article/10.3390/medicina60010071/s1, Table S1: Demographics and clinical parameters profile of HFNC survivors and CPAP failure; Table S2: Laboratory findings profile according to the respiratory support; Table S3: Comparison of respiratory parameters between HFNC survivors and c-PAP failure.
