# Supplementary material for: Stratification of COVID-19 Patients with Moderate-to-Severe Hypoxemic Respiratory Failure for Response to High-Flow Nasal Cannula: A Retrospective Observational Study
Source: Medicina (Kaunas). 2023 Dec 29;60(1):71. doi: 10.3390/medicina60010071 (PMC10819134; doi:10.3390/medicina60010071)
Supplement: Supplementary file 1 [file medicina-60-00071-s001.zip › medicina-2631128-supplementary.pdf]

Table S1. Demographics and clinical parameters profile of HFNC survivors and CPAP failure.

	HFNC survivors (n=64)	CPAP failure (n=41)	*p*
Demographics			
Age	69 (59 - 75)	74 (70 - 79)	**0.001**
BMI	27.4 (24.2 - 29.4)	27.3 (24.5 - 29.3)	0.892
Gender, male	40 (62.5)	21 (51.2)	0.254
Hospital stay (days)	21.5 (14.25 - 31.5)	13 (9 - 17.75)	**0.001**
ICU admission (28 days)	3 (4.62)	25 (60.9)	**0.001**
COVID-19 Vaccination	13 (20.3)	7 (17.5)	0.762
Comorbidities	*3 (2 - 5)*	4 (3 - 6)	0.101
Charlson Comorbidity Index	*3 (2 - 5)*	5 (4 - 6)	0.007
Diabetes	33 (51.1)	18 (43.9)	0.473
Coronary artery disease	18 (28.1)	13 (31.7)	0.694
COPD	14 (20.3)	6 (14.6)	0.461
Heart failure	15 (23.4)	15 (36.5)	0.148
Hypertension	37 (57.8)	32 (78)	0.081
Chronic kidney disease	7 (10.9)	9 (21.9)	0.261
Atrial fibrillation	5 (7.8)	8 (19.5)	0.077
Cerebrovascular disease	2 (6.4)	3 (7.3)	0.335
Respiratory failure	0 (0)	0 (0)	-
Vital and clinical parameters			
Heart rate	80 (70 - 95)	82 (76 - 92)	0.338
DBP mmHg	80 (65 - 85)	75 (70 - 83.75)	0,732
SBP mmHg	132.5 (120 - 148)	135 (130 - 150)	0.213
MBP mmHg	94 (83 - 106)	95 (88 - 101)	0.626
Fever	28 (43.7)	22 (53.6)	0.324
Glasgow Coma Scale	15 (15 - 15)	15 (15 - 15)	0.665
Therapy			
Corticosteroids	64 (100)	41 (100)	1
Anti-IL6	8 (12.5)	7 (17)	0.522
Remdesivir	1 (1.5)	1 (2.4)	0.739
Anti-SARS-CoV-2	1 (1.5)	3 (7.3)	0.129

Data are n (%) and median (IQR). N is the total number of patients with available data.

BMI: Body mass index; ICU: Intensive care Unit; COPD: Chronic Obstructive pulmonary disease; COVID-19: coronavirus disease 2019; DBP: diastolic blood pressure; IL-6: interleukin 6; SBP: systolic blood pressure; MBP: mean blood pressure; IL-6: interleukin 6 ICU: Intensive care Unit; MBP: mean blood pressure; SARS-CoV-2: Severe acute respiratory syndrome coronavirus 2; SBP: systolic blood pressure

Table S2. Laboratory findings profile according to the respiratory support .

	**HFNC survivors (n=64)**	**CPAP failure (n=41)**	***p***
Laboratory findings			
Albumin, *g/dL*	3.14 (2.9 – 3.3)	2.9 (2.7 – 3.28)	**0.024**
ALT, *UI/L*	25 (15 - 42)	30 (20 - 47)	0.257
AST, *UI/L*	29 (21 - 40)	30 (23 - 66)	0.449
CK, *U/L*	100 (51 - 210)	103 (57 - 266)	0.537
Creatinine, *mg/dL*	1 (1 - 1)	1 (1 - 2)	0.264
D-dimer, *mcg/mL*	3 (3 - 4)	4 (4 - 4)	0.320
Fibrinogen, *mg/dL*	640 (462 – 761)	607 (533 - 682)	0.182
Hb, *gr/dL*	14 (12 - 14)	14 (11 - 15)	0.884
IL-6, *pg/mL*	28 (10 – 66.8)	63.9 (40 - 113)	**0.001**
LDH, *U/L*	351 (281 - 436)	438 (343 - 608)	**0.011**
Bilirubin, total, *mg/dl*	0,65 (0,44 – 0,84)	0,64 (0,5 – 1)	0.151
NT-proBNP, *pg/mL*	497 (183 - 1625)	641 (229 - 2518)	0.510
CRP, *mg/dL*	6.3 (3.3 – 14.3)	10.3 (5.2 - 12.9)	0.293
PCT, *ng/mL*	0.14 (0.08 – 0.46)	0.15 (0.08 – 0.44)	0.956
Ferritin, *mg/dl*	652 (254 – 2170)	743 (471 – 1358)	0.509
Platelet count, *cells x* 10^4	223 (166 - 289)	198 (150 - 246)	0.216
Troponin, *pg/mL*	30.3 (16 - 85)	54 (14.6 – 116.1)	**0.006**
Urea, *mg/dL*	56 (41 – 84.5)	55 (39 - 82)	0.774
WBC, *cells*	7245 (4900 - 12300)	9100 (6200 - 12100)	0.169
Lymphocyte count, *cells*	803 (599 - 1219)	815 (560 - 1090)	0.351
Neutrophil count, *cells*	5748 (3425 – 9477)	7280 (4836 – 10164)	0.299
Neutrophil/lymphocyte ratio	7.4 (4.1 – 10)	8.5 (5.7 – 14)	**0.049**

Data are n (%) and median (IQR). N is the total number of patients with available data.

ALT: Alanine transaminase; AST: aspartate transaminase; CK: creatine kinase; CRP: C-reactive protein; Hb: hemoglobin; IL-6: interleukin 6; LDH: lactate dehydrogenase; NT-proBNP: N-terminal pro-brain natriuretic peptide; PCT: procalcitonin; WBC: white blood cells

Table S3. Comparison of respiratory parameters between HFNC survivors and c-PAP failure.

	HFNC survivors (n=64)	c-PAP failure (n=41)	*p*
Respiratory Rate, T0	20 (20 - 25)	25 (21 - 30)	**0.004**
SpO_2/FiO_2, T0	158.3 (153.3 - 187)	153.3 (147 - 201.2)	0.243
ROX, T0	7.5 (6.8 - 8.7)	6.2 (4.9 - 8.5)	0.075
PaO_2/FiO_2, T0	113.5 (97.8 - 133.33)	99.1 (90.4 - 136.2)	0.152
Respiratory Rate, T6	20 (20 - 22)	24 (21 - 27)	**0.001**
SpO_2/FiO_2, T6	163 (160 - 171)	158.3 (153 - 163)	**0.008**
ROX, T6	8 (7.1 - 8.7)	6.4 (5.3 - 7.7)	**0.001**
PaO_2/FiO_2, T6	138.6 (128.3 - 166)	128.3 (110 - 184)	0.098
Respiratory Rate, T24	20 (18 - 22)	22 (20 - 26)	**0.005**
SpO_2/FiO_2, T24	174 (160 - 196)	157 (135 - 163)	**0.001**
ROX, T24	8.9 (7.7 - 10.5)	7.2 (5.8 - 8.8)	**0.001**
PaO_2/FiO_2, T24	151 (120 - 176)	116 (85 - 156)	**0.005**
Respiratory Rate, T48	20 (18 - 22)	21 (20 - 24)	**0.047**
SpO_2/FiO_2, T48	165 (161.6 - 206.6)	160.6 (156.6 - 180)	0.071
ROX, T48	8.9 (7.9 - 10.4)	7.6 (6.4 - 9)	**0.007**
PaO_2/FiO_2, T48	148 (129 - 168)	116 (103 - 130)	**0.001**

Data are n (%) and median (IQR). N is the total number of patients with available data.

FiO2: fraction of inspired oxygen; PaCO2: partial pressure of carbon dioxide; PaO2: partial pressure of oxygen; ROX: respiratory rate oxygenation; SO2: oxygen saturation
